# 3D printed polylactic acid - hemp fiber composites: Mechanical, thermal, and microcomputed tomography data

**DOI:** 10.1016/j.dib.2021.107534

**Published:** 2021-11-01

**Authors:** John Arnold, Damon A. Smith

**Affiliations:** a Department of Mechanical Engineering, University of New Orleans, United States; b Advanced Materials Research Institute (AMRI), University of New Orleans, United States

**Keywords:** Hemp, Additive manufacturing, 3D printing, Green manufacturing, Biomaterials

## Abstract

Hemp fiber was used untreated and treated with sodium hydroxide or (3-aminopropyl)triethoxysilane (APTES) as an additive in polylactic acid (PLA) for fused filament fabrication (FFF) of tensile test specimens. Composites granules were produced by solvent processing with 10 wt. % of hemp fiber to use as feedstock for the extrusion of filaments compatible with commercial FFF printers. The dataset shows the thermal properties of the various composites, which were used to determine the appropriate temperatures required for extrusion of filaments and FFF printer settings. Microcomputed tomography imaging was performed and tensile mechanical properties of FFF-printed tensile test specimens were determined as a function of hemp fiber surface treatment. The data provides an assessment of the use of minimally processed hemp fiber as a filler or mechanical enhancer of thermoplastic materials for additive manufacturing.

## Specifications Table

**Table:** 

Subject	Chemistry, Materials Science, Manufacturing Engineering
Specific subject area	Hemp, Polylactic acid, Composite, 3D Printing, Fused Deposition Modeling, Fused Filament Fabrication, Additive Manufacturing
Type of data	Table, Image, Graph, Figure
How data were acquired	Differential scanning calorimetry (DSC) (TA Instruments SDT q600 TGA-DSC), Optical microscopy (40x-1500x Infinity Kohler Plan Inverted Microscope with 18MP Camera), Digital camera (Elmo-12F), Microcomputed tomography (MicroCT) (Xradia microXCT 400 Scanner), Tensile mechanical tester (MTI Instruments SEMtester 2000)
Data format	Raw, Analyzed
Parameters for data collection	Hemp fiber was used as-is or treated with sodium hydroxide or (3-aminopropyl)triethoxysilane (APTES). Hemp fiber and polylactic acid were dissolved in chloroform and the solutions were dried in a vacuum oven followed by mechanical grinding prior to thermal characterization and extrusion into filaments. The filaments were used as feedstock to print tensile test specimens by fused filament fabrication (FFF).
Description of data collection	Optical microscope images of the untreated hemp fiber were acquired at 40x magnification. DSC data for 10 mg of composite granules with no hemp fiber, untreated hemp fiber, and treated hemp fiber were collected at a rate of 10°C min^−1^ in a nitrogen atmosphere. MicroCT was performed on a FFF-printed tensile test specimen using a 4X objective at 120 kV, a power of 10 W, and a 3.5 s acquisition time. MicroCT images were reconstructed with an isotropic resolution of 5 μm. Stress-strain data was acquired at room temperature at a rate of 6 mm min^−1^ with data collected at 10 Hz using a 9 kN load cell.
Data source location	University of New Orleans, New Orleans, LA, USA
Data accessibility	All raw data are available in this article and the following repository: https://data.mendeley.com/datasets/f2jt9sfxmf/1

## Value of the Data

• This data is useful for understanding the influence of minimally process hemp fiber with varying surface treatments on the thermal and mechanical properties of PLA - hemp fiber composites printed by fused filament fabrication (FFF).
• Researchers and manufacturers can use this data as a baseline for determining the necessary processing and treatment requirements for thermoplastic materials with hemp fiber additives.
• They can be reused and combined with additional composite formulations and testing dependent on future targeted physical properties for manufactured objects.

## 1 Data Description

**Fig. 1:**
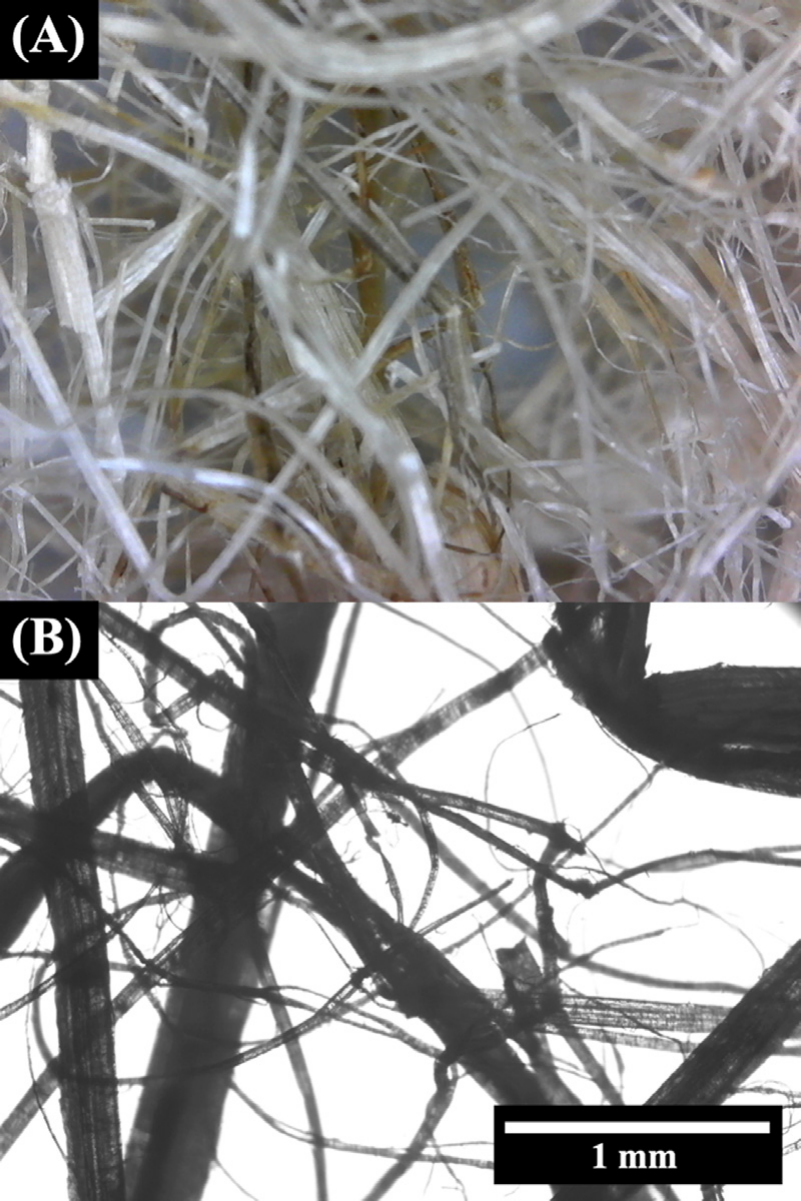
(A) Digital photo and (B) optical microscope image of unprocessed hemp fiber.

Fig. 1 shows a representative digital photo and optical microscope image of the unprocessed hemp fiber used in the acquisition of this data. The hemp fiber was purchased with an average length of one inch, as determined by the supplier. The average diameter of hemp fiber bundles was 56 ± 65 μm, determined from 100 measurements from 6 optical microscope images. Additional optical microscope images are available in the data repository.

**Fig. 2:**
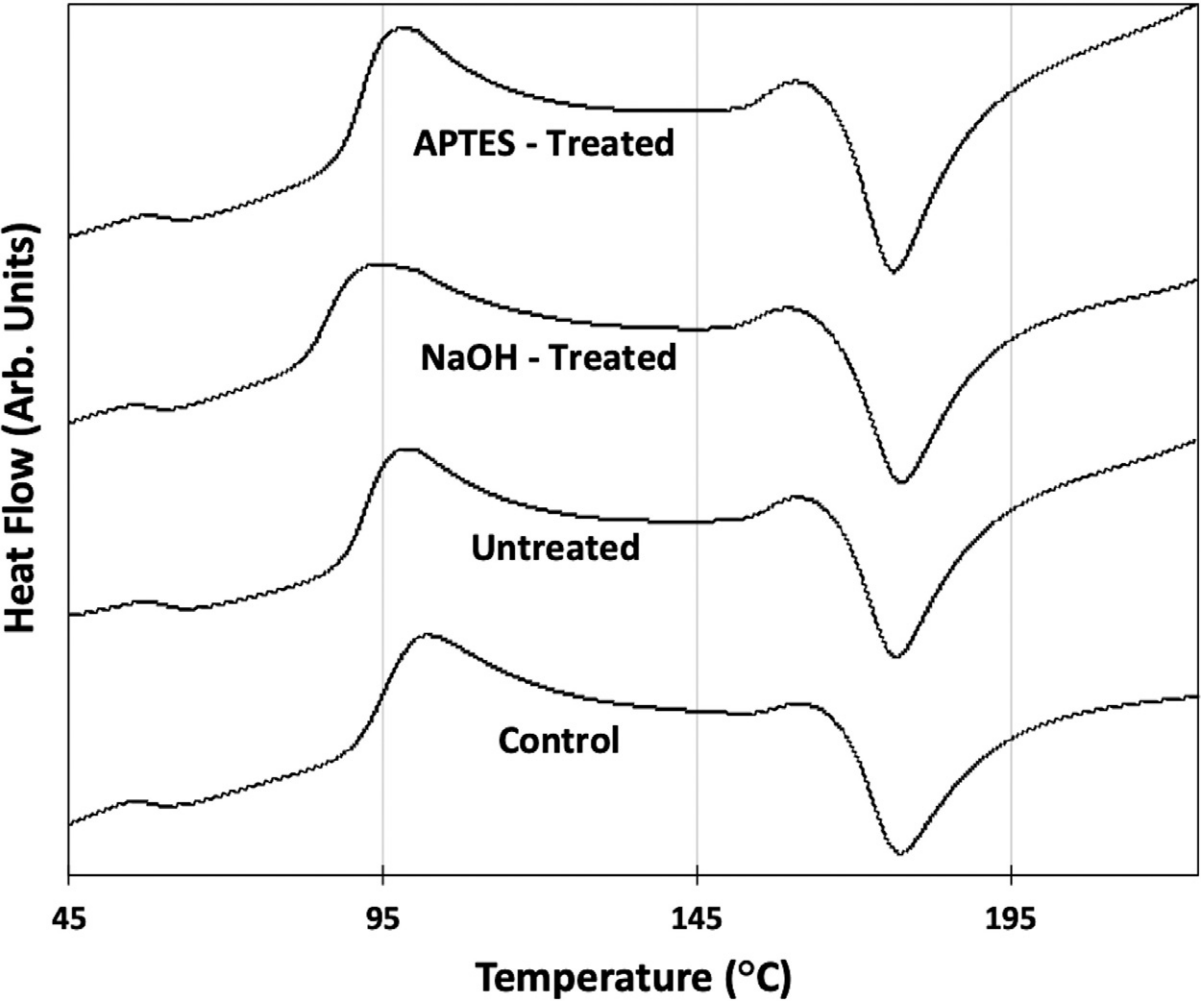
Differential scanning calorimetry (DSC) data of PLA - hemp fiber composite granules with different surface treatments compared to a control specimen consisting of PLA with no additives.

Normalized and shifted differential scanning calorimetry (DSC) data for PLA granules produced with and without 10 wt. % of hemp fiber additives with no surface treatment, sodium hydroxide (NaOH) treatment, or (3-aminopropyl)triethoxysilane (APTES) treatment are shown in Fig. 2. This data was collected to establish the glass transition temperature, T_G_, and the melting point, T_M_, of the control and composite granules for determination of the appropriate temperatures for fused filament fabrication (FFF) printing of tensile test specimens. Table 1 summarizes the glass transition temperature, T_G_, and the melting point, T_M_, for the various granules extracted from the DSC data in Fig. 2. DSC of the granules showed only a moderate change in the T_G_ and T_M_ with the addition of the hemp fiber additives.

**Table 1:** Glass transition temperature, T_G_, and the melting point, T_M_, of composite granules with different surface treatments and the control specimen extracted from DSC data.

*Specimen*	*Glass Transition Temperature (°C)*	*Melting Point (°C)*
***Control***	58.4	177.4
***Untreated***	60.5	176.8
***NaOH - Treated***	58.2	177.5
***APTES - Treated***	60.3	176.4

**Fig. 3:**
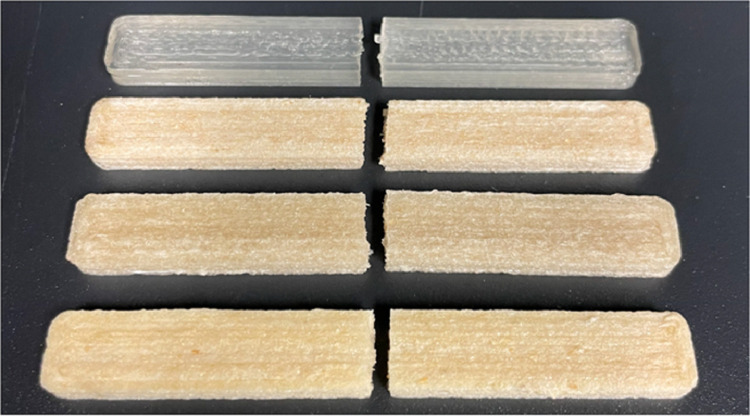
Photo of representative fractured FFF-printed tensile test specimens comprised of (from top to bottom) PLA and PLA containing 10 wt. % hemp fiber that is untreated, NaOH-treated, and APTES-treated.

A digital photo of representative fractured FFF-printed tensile test specimens comprised of PLA or PLA containing 10 wt. % of hemp fiber is shown in Fig. 3, produced using extruded filament from the composite granules as feedstock. Hemp fiber additives were used in the composites that were untreated or treated with NaOH or APTES. Specimen geometries were based on a slight modification of the Type I specimens defined in the American Society for Testing and Materials (ASTM) D638-14 standard test methods for determining the tensile properties of plastics.

**Fig. 4:**
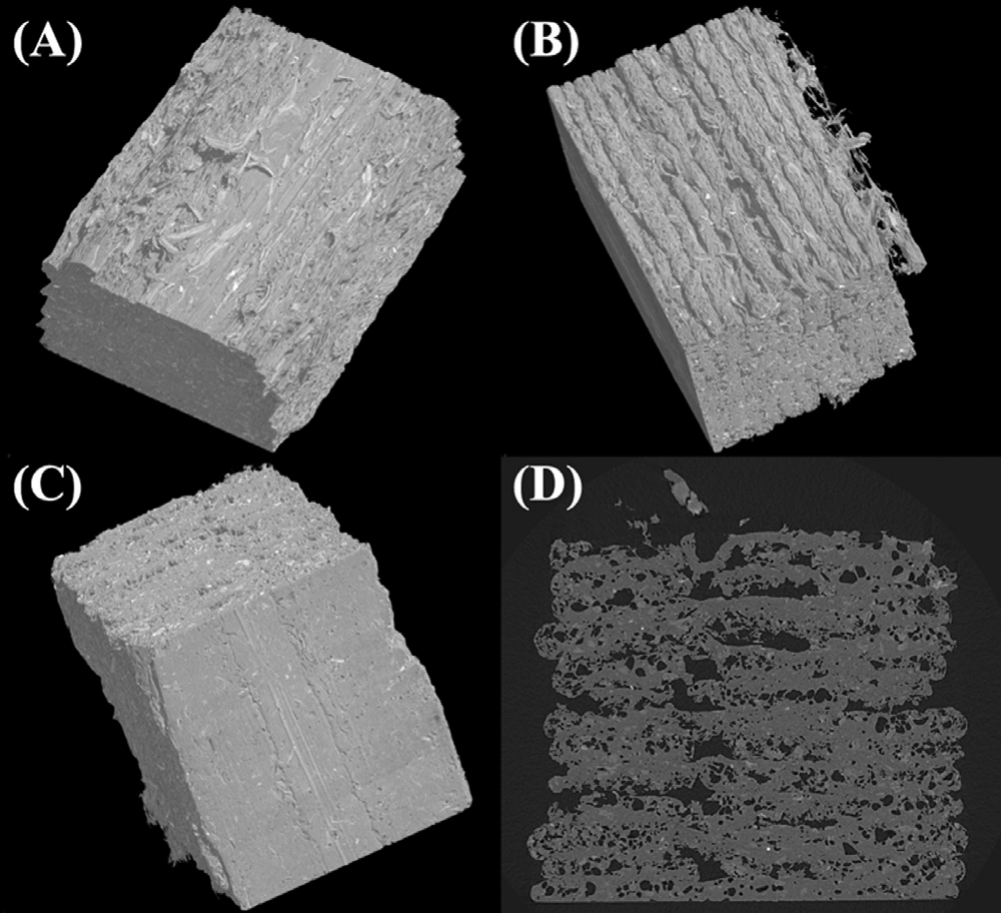
Microcomputed tomography data of a representative PLA - hemp fiber tensile test specimen printed by fused filament fabrication. Three-dimensional representations are displayed showing the (A) top, (B) side, and (C) bottom of the specimens. A (D) two-dimensional slice is also shown which displays the porous structure of the specimen represented by the low contrast regions.

Fig. 4 shows microcomputed tomography (microCT) images of the middle section of a representative PLA - hemp fiber tensile test specimen with APTES surface treatment. The three-dimensional (3D) reconstruction shows the different surface roughness on the top (Fig. 4A), side (Fig. 4B), and bottom (Fig. 4C) of the printed specimen. The smooth surface of the bottom of the specimen resulted from the deposition onto the borosilicate glass build platform of the FFF printer. A two-dimensional (2D) slice through the specimen cross-section displays significant porosity represented by the regions of low contrast (Fig. 4D).

**Fig. 5:**
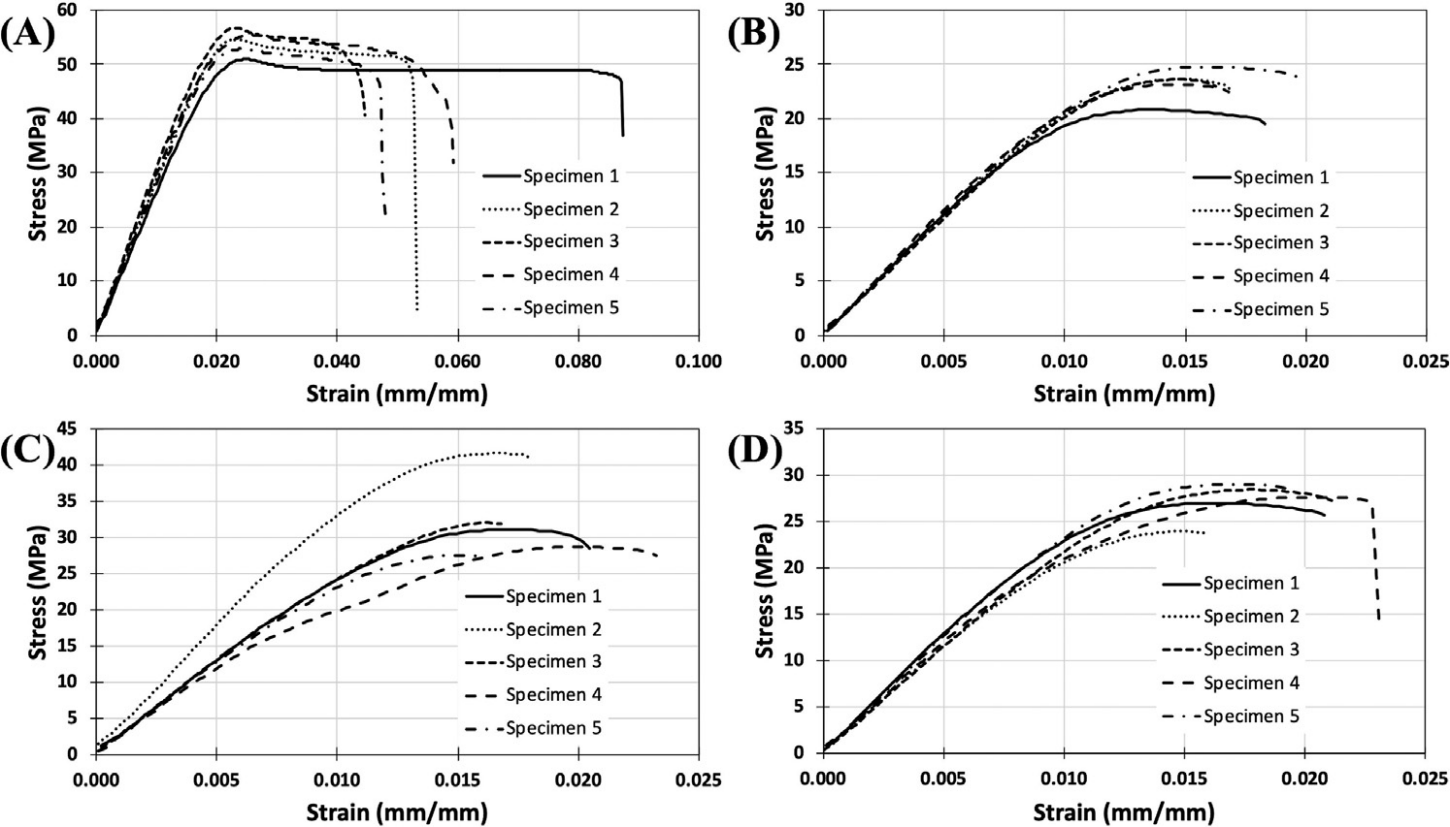
Tensile mechanical testing data for the (A) control specimens and PLA - hemp fiber composites with (B) untreated hemp fiber, (C) sodium hydroxide-treated hemp fiber, and (D) APTES-treated hemp fiber.

**Fig. 6:**
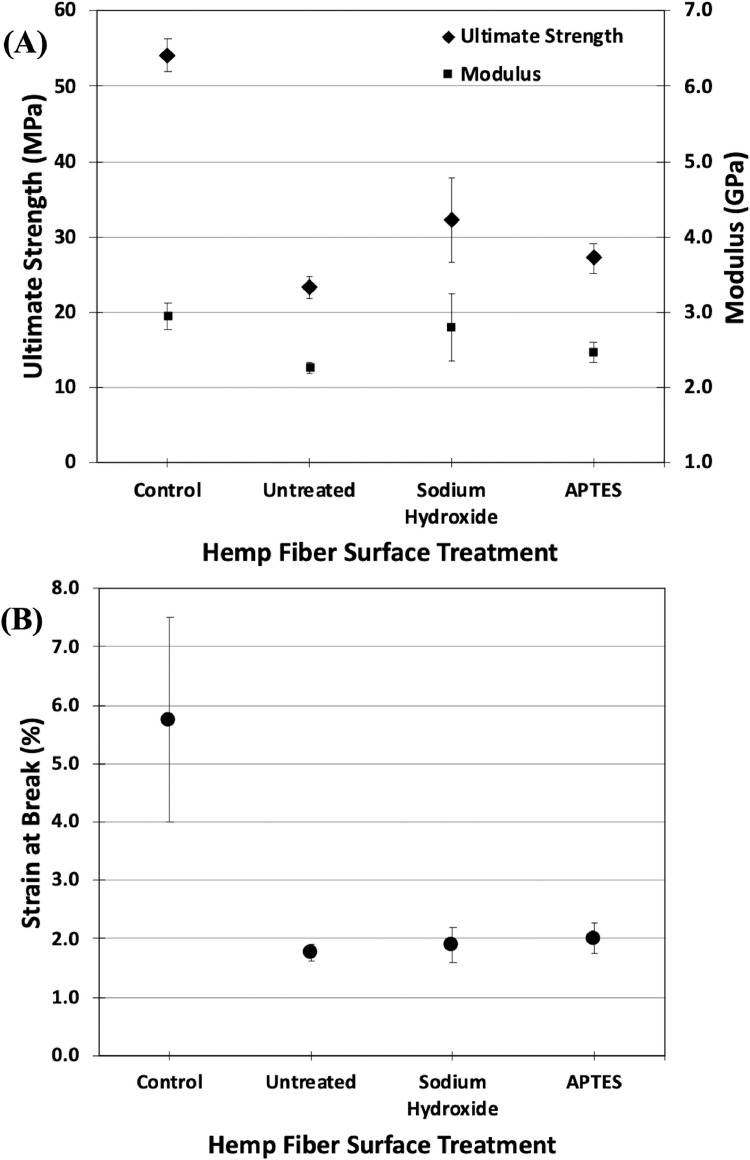
The average (A) ultimate strength, modulus, and (B) strain at break of control and PLA - hemp fiber tensile test specimens with varying surface treatments. Error bars indicate the standard deviation.

Stress-strain curves for FFF-printed tensile test specimens consisting of PLA with and without hemp fiber additives are shown in Fig. 5. The average ultimate strength, modulus, and strain at break for each specimen type was extracted from the stress-strain data and is displayed in Fig. 6. The data shows a major reduction in the average ultimate strength and strain at break for the PLA - hemp fiber specimens compared to the control specimens, due to the porosity observed in Fig. 4D. Some slight variation in the modulus was observed amongst the different sample sets. The NaOH-treated composites showed the highest average ultimate strength and modulus. Single factor ANOVA analysis at the p < 0.05 level showed statistically significant variation for ultimate strength (F(3, 16) = 90.05, p = 3.109e-10), modulus (F(3, 16) = 7.40, p = 0.002), and strain at fracture (F(3, 16) = 35.91, p = 2.458e-7). Statistical significance was also found for the ultimate strength (F(2, 12) = 8.11, p = 0.006), modulus (F(2, 12) = 4.85, p = 0.029), and strain at fracture (F(2, 12) = 3.92, p = 0.049) when comparing only the PLA - hemp fiber composites.

## 2 Experimental Design, Materials and Methods

### 2.1 Processing and fused filament fabrication of polylactic acid - hemp fiber composites

Hemp fiber with an average length of approximately one inch was purchased (American Hemp, LLC, USA) that was previously processed by a single mechanical decortication. The received hemp fiber was rinsed with deionized water and dried in a vacuum oven for 24 h at 40 °C. The hemp fiber was then used as-is or was further treated with NaOH or APTES. Sodium hydroxide treatment was performed by adding hemp fiber to a 5 wt. % solution of NaOH in deionized (DI) water. The solution was stirred in an incubator stirrer for 24 h at a rate of 150 rpm and a temperature of 40 °C. The solution was then neutralized using glacial acetic acid, rinsed with DI water, and then dried in a vacuum oven for 24 h at 40 °C. Surface treatment of hemp fiber by ATPES was performed by adding the NaOH-treated fiber to a 1:4 ratio solution of ethanol and DI water containing 0.2 vol. % of APTES. The solution was stirred for 24 h at 150 rpm and 40 °C prior to rinsing with DI water and drying under vacuum for 24 h at 40 °C.

Composite granules were produced using a solvent-casting method for extrusion of filaments compatible with FFF printing systems, similar to previously reported methods [1], [2], [3]. Composite granules were made with 10 wt. % of hemp fiber that was untreated, treated with NaOH, and treated with NaOH followed by APTES. Control granules were created consisting of PLA without any additives to account for any mechanical or thermal property variation due to the solvent-casting process. For each of the granules produced, 90 g of PLA pellets were added to 500 mL of chloroform and stirred at 300 rpm at a temperature of 60°C until the pellets were dissolved. 90 g of hemp fiber was added to the bottom of a glass dish and the PLA - chloroform solution was poured over the fiber. The solution was allowed to dry under ambient conditions for 48 h followed by removal of residual solvent in a vacuum oven at 40 °C for 24 h. The composite was then granulated (Minigran, Dynisco) until it could pass through a 5 mm screen. The granulated composite was dried for an additional 24 h under vacuum at 40°C prior to extrusion.

The PLA and PLA - hemp fiber granules were extruded using a single screw extrusion system (EX2, Filabot) at a temperature 5 °C above the T_M_ of the composites determined by DSC. The composite was extruded through a 1.75 mm circular die at a rate of 150 cm min^−1^, which then passed through an air bath (Airpath, Filabot). The extruded filaments were used as feedstock for printing tensile test specimens using a Fusion3 F400-S FFF system. Test specimens were printed using a 1 mm nozzle size with nozzle and build platform temperatures of 215 °C and 60 °C, respectively. The specimens were also printed with 2 outer perimeters and a 100 % rectilinear infill. The perimeters and infill regions were printed at a speed of 50 and 200 mm s^−1^, respectively. Specimens were store in a humidity-controlled cabinet at 20 % RH for 24 h prior to tensile testing with no additional processing.

### 2.2 Characterization

Optical microscope images of the hemp fiber were acquired at 40x magnification using a 40x-1500x Infinity Kohler Plan Inverted Microscope with 18MP Camera. Measurements of the hemp diameter was performed by analysis of the optical microscope images using imageJ software and a random sample of 100 fibers. DSC (SDT Q600 TGA-DSC, TA Instruments) was performed in a nitrogen atmosphere. The T_G_ and T_M_ of the control and composite granules were extracted from DSC curves taken from temperatures of 45 to 225 °C at a rate 10 °C min^−1^. MicroCT images were acquired on an Xradia microXCT 400 scanner using a 4X objective at 120 kV, a power of 10 W, and a 3.5 s acquisition time. Images were reconstructed with an isotropic resolution of 5 μm. Tensile testing was performed on 5 test specimens of each type that were printed with a slight variation of the Type I geometry based on the ASTM D638-14 standard test method for determining the tensile properties of plastics. The stress-strain data was acquired at room temperature on an MTI Instruments SEMtester 2000 with a 9 kN load cell. Stress was applied at a rate of 6 mm min^−1^ with data collected at 10 Hz.

## Ethics Statement

This work did not involve human subjects, animal experiments, or data collected from social media platforms.

## CRediT authorship contribution statement

**John Arnold:** Conceptualization, Methodology, Formal analysis. **Damon A. Smith:** Supervision, Conceptualization, Writing – review & editing, Funding acquisition, Project administration.

## Declaration of Competing Interest

The authors declare that they have no known competing financial interests or personal relationships which have or could be perceived to have influenced the work reported in this article.
